# An overview on the recently discovered iota-carbonic anhydrases

**DOI:** 10.1080/14756366.2021.1972995

**Published:** 2021-09-06

**Authors:** Alessio Nocentini, Claudiu T. Supuran, Clemente Capasso

**Affiliations:** a Department of NEUROFARBA, Section of Pharmaceutical and Nutraceutical Sciences, University of Florence, Firenze, Italy; b Department of Biology, Agriculture and Food Sciences, Institute of Biosciences and Bioresources, CNR, Napoli, Italy

**Keywords:** Carbonic anhydrases, CA classes, hydratase activity, bacteria, catalytic pocket

## Abstract

Carbonic anhydrases (CAs, EC 4.2.1.1) have been studied for decades and have been classified as a superfamily of enzymes which includes, up to date, eight gene families or classes indicated with the Greek letters α, β, γ, δ, ζ, η, θ, ι. This versatile enzyme superfamily is involved in multiple physiological processes, catalysing a fundamental reaction for all living organisms, the reversible hydration of carbon dioxide to bicarbonate and a proton. Recently, the ι-CA (LCIP63) from the diatom *Thalassiosira pseudonana* and a bacterial ι-CA (BteCAι) identified in the genome of *Burkholderia territorii* were characterised. The recombinant BteCAι was observed to act as an excellent catalyst for the physiologic reaction. Very recently, the discovery of a novel ι-CAs (COG4337) in the eukaryotic microalga *Bigelowiella natans* and the cyanobacterium *Anabaena sp.* PCC7120 has brought to light an unexpected feature for this ancient superfamily: this ι-CAs was catalytically active without a metal ion cofactor, unlike the previous reported ι-CAs as well as all known CAs investigated so far. This review reports recent investigations on ι-CAs obtained in these last three years, highlighting their peculiar features, and hypothesising that possibly this new CA family shows catalytic activity without the need of metal ions.

## 1. Introduction

CAs represent a superfamily of enzymes considered among the most versatile on the planet. They are ubiquitously present in Archaea, Bacteria, and Eukaryote domains, with the function to accelerate a fundamental reaction for all living organisms, the reversible hydration of carbon dioxide (CO_2_) to bicarbonate (HCO_3_^-^) and a proton (H^+^), according to the following chemical reaction: CO_2_ + H_2_O ⇋ HCO_3_^-^ + H^+^^1–7^. The reversible spontaneous CO_2_ hydration reaction occurs with a rate of 0.15 s ^− 1^ that cannot meet the fast demand of CO_2_ and HCO_3_^-^ necessary for a complex sequences of metabolic pathways, which allow organisms to grow and reproduce, maintain their structures, and respond to environmental changes^6^^,^^7^. CA activity increases the velocity of the CO_2_ hydration reaction from 100,000 to one million times per second (k_cat_ falling in the range of 10^4^–10^6^ s ^− 1^) with respect to the uncatalyzed reaction, making this superfamily of enzymes among the fastest biocatalysts known in nature^7^.

CAs are involved in multiple physiological processes in all organisms in which they are present, such as respiration, photosynthesis, CO_2_ and bicarbonate transport, pH and CO_2_ homeostasis, electrolyte secretion in various tissues and organs, bone resorption, and calcification, etc.^8^. For example, carbon dioxide is a byproduct of sugar and fat breakdown in cells and needs to be removed from the mammalian cells^9^. At the level of peripheral tissues, the CO_2_ produced by cellular aerobic metabolism leaves the cells and enters the bloodstream by a pressure gradient effect. Approximately 90% of the CO_2_ flows into red blood cells and is converted to bicarbonate by CAs^9^. The produced bicarbonate leaves the red blood cells via an anion exchanger (AE) protein and is transported from the bloodstream to the lungs. At the alveolar level, the concentration of CO_2_ is lower than in peripheral tissues, whereas there is a higher concentration of bicarbonate, which is pumped into the red blood cell^9^. Here, through the action of the reverse reaction catalysed by CA, bicarbonate is transformed into water and CO_2_. The CO_2_ produced in this way is released into the bloodstream and, passing through the alveolus walls, is exhaled. Among others, gluconeogenesis, lipogenesis, and ureagenesis are several biosynthetic reactions that use pyruvate carboxylase (PC), acetyl-Co-A carboxylase (ACC), and carbamoyl-phosphate synthetase I and II, respectively, also use bicarbonate as substrate for carboxylation reactions^8^^,^^10–12^. The bicarbonate is produced in a CA-dependent manner.^10–12^

In plants, CO_2_ is stored as bicarbonate ions. In both terrestrial and aquatic plants, CA converts HCO_3_^-^ ions to CO_2_, which is concentrated in the proximity of the enzyme RuBisCO (Ribulose Bisphosphate Carboxylase/Oxygenase) present in the stroma of the chloroplasts^13–15^. As a result, the performance of RuBisCO carboxylation reaction is increased, whereas its oxygenation is suppressed. Eukaryotic unicellular photosynthetic organisms have evolved diverse Carbon Concentrating Mechanisms (CCMs) to increase CO_2_ concentration in the proximity of RuBisCO up to 1000-fold from the low CO_2_ levels present in the environment. In algae, the main component of the CCM is the pyrenoid^16^^,^^17^. In cyanobacteria, the equivalent of the pyrenoid is the carboxysome. Carboxysomes are composed of RuBisCO, CAs, active bicarbonate transporters, and structural envelope proteins^18^. The structure of the carboxysome envelope prevents the escape of CO_2_ from these organelles.

Another notable biological phenomenon in which CAs are involved is represented by coral calcification^19–23^. Calcium in seawater reacts with the HCO_3_^-^ produced by coral CAs to form calcium carbonate and protons, which are extruded. CaCO_3_ is thereafter deposited and generates the hard outer surface of corals.^19^

In bacteria, the CA catalysed reaction is the only known pathway to obtain and balance endogenous levels of CO_2_, H_2_CO_3_ (carbonic acid), HCO_3_^-^, and CO_3_^2-^(carbonate) rapidly^7^^,^^24–26^. In bacteria, CO_2_ enters and leaves the bacterial cell by passive diffusion, while bicarbonate is imported directly into the cell through bicarbonate transporters^27^. Gram-negative bacteria have a periplasmic CA in their periplasmic space, for avoiding the loss of CO_2_ through diffusion. This enzyme converts faster the CO_2_ generated from the bacterial metabolism and that coming from the atmosphere into bicarbonate. HCO_3_^-^ is thereafter pumped into the cytoplasm by bicarbonate transporters and, there, converted into CO_2_ by cytoplasmic forms of CAs belonging to the β- and/or γ-CA classes^15^^,^^24^^,^^27^. Thus, the bicarbonate transporters and bacterial CA enzymes provide CO_2_ and HCO_3_^-^ to sustain bacterial metabolism^15^^,^^24^^,^^27^. The natural reaction of interconversion of CO_2_ and H_2_O into HCO_3_^-^ and H^+^ cannot quickly supply CO_2_ and HCO_3_^-^ to the bacterial metabolism, as already mentioned, since the reaction rate is too low at physiological pH.

From these examples, it is readily apparent the enzyme versatility of CAs, which are considered metabolic enzymes involved in many physiological processes indispensable for the lifecycle of most living organisms^7^^,^^25^.

The CA superfamily includes, up until now, eight gene families or classes indicated with the letters of the Greek alphabet (α, β, γ, δ, ζ, η, θ, ι)^1–5^. The distribution of the CA classes is somewhat assorted in most investigated organisms, and except for mammals which encode only for α-CAs, most of them possess multiple representatives of two or even more genetic families. The genome of mammals encodes only for the α-CA class, of which 15 isoforms have been identified^8^^,^^28–31^. In plants, α and β-CAs have been recognised^32^. In Bacteria, Archaea, and cyanobacteria are present α, β, γ, and ι -CA classes^5–7^^,^^32–34^. Marine diatoms encode for α- δ-, ζ-, θ- and ι-CAs^35–37^. In protozoa have been detected α- β and η-CAs. Probably, the η-CA-class, recently discovered, has a pivotal role in *de novo* purine/pyrimidine biosynthetic pathways in these organisms^38^. In the fungal kingdom, the typical class is represented by β-CAs, and most fungi encode at least one β-CA^39–41^. In contrast, most filamentous ascomycetes contain multiple β-CA genes and, in some of them, it is possible to also find genes encoding for α-CAs^39–41^.

The eight CA classes are phylogenetically unrelated and, thus, they can be classified as non-homologous isofunctional enzymes that catalyse the same reaction^1–7^. This is an example of convergent evolution since CA classes show low sequence similarity in primary and possibly tertiary structures because they evolved in a different biological contexts, but catalysing the same reaction, with the active site residues showing a rather similar geometry. As mentioned above, CAs are metalloenzymes whose catalytic site contains a metal ion cofactor necessary for enzyme catalysis^5–7^^,^^34^. Usually, the Zn^2+^ ion cofactor is coordinated by three amino acid residues, which may be three His residues in the α-, γ-, δ- and, probably, θ-classes; one His, and two Cys residues in β- and ζ-CAs, and two His and one Gln residues in the η-class^42^. Simultaneously, the fourth ligand is a water molecule/hydroxide ion acting as the nucleophile in the catalytic enzyme cycle^5^^,^^6^^,^^28^^,^^34^^,^^43^^,^^44^. Some CA-classes can also coordinate metal ions different from Zn^2+^, such as Co^2+^, Cd^2+^, Fe^2+^, and Mn^2+^. As described in the literature, α-, β-, δ-, η- and, perhaps θ-CAs use as ion cofactor the Zn^2+^; γ-CAs the Fe^2+^, although they can coordinate Zn^2+^ or Co^2+^, too^31^^,^^45–51^. The ζ-CAs are active with either Cd^2+^ or Zn^2+^incorporated into the same apoprotein and are defined as cambialistic enzymes^52–54^. From a structural point of view, the representative belonging to one CA-class shows a different folding and structure compared with those of other CA-classes. α-CAs are usually active as monomers or dimers; β-CAs are active only as dimers, tetramers, or octamers. The γ-CAs must be trimers for accomplishing their catalytic function^46^^,^^47^^,^^50^^,^^55^. γ-CA monomers are characterised by a tandemly-repeated hexapeptide, which is crucial for the left-hand fold of the trimeric β-helix structures^56^. The X-ray structure of the θ-CAs resulted to be very similar to some β-CAs^57^. The crystal structure of ζ-CA showed three slightly different active sites on the same polypeptide chain^54^. No information is available so far on the structural organisation of δ- and η-CAs. Intriguingly, α-, η-, θ- and ι-CAs were reported to catalyse the esters/thioesters hydrolysis, while no esterase activity was detected for the other CA families^28^^,^^58^^,^^59^.

## 2. The ultimately discovered class, the ι-CA

### 2.1. Lcip63 and BteCAι

In 2019 Gontero et al. discovered the ι-CAs (acronym LCIP63) by exploring the genome of the diatom *Thalassiosira pseudonana*^59^. LCIP63 was stated to prefer as ion cofactor Mn^2+^ to Zn^2+^, being localised in the chloroplast, and being only expressed at low concentrations of CO_2_, confirming their primary role in the diatom CCM.^59^ These authors also reported LCIP63 homologs in the genome of other diatoms and algae, bacteria, and archaea. Most of the LCIP63 homologs identified in bacteria have been annotated in the data bank as SgcJ/EcaC oxidoreductase family with an unknown function^59^. In 2020, Capasso et al. demonstrated that the recombinant bacterial ι-CA (acronym BteCAι) identified in the genome of *Burkholderia territorii* resulted to be excellent catalyst for the hydration of CO_2_ to bicarbonate and protons with a k_cat_ of 3.0 × 10^5^ s ^− 1^ and k_cat_/K_M_ of 3.9 × 10^7^ M ^− 1^ s ^−1^^60^. Addition of Zn^2+^ or Ca^2+^ to the culture media for enzyme expression in *E. coli* allowed catalytically active enzyme. In contrast, by adding Mn^2+^, the enzyme activity was not present or the enzyme was found to contain zinc, probably from the traces of this ion present as impurity in the used reagents^60^. The protein resulted sensitive to inhibition with substituted benzene-sulphonamides and clinically licenced sulfonamide-, sulfamate- and sulfamide-type drugs, which are among the most investigated CA inhibitors (CAIs)^61^. BteCAι inhibition profile showed several benzene-sulphonamides with an inhibition constant lower than 100 nM^61^. In addition to sulphonamides and their bioisosteres, anion and small molecules (another group of CAIs) were investigated as BteCAι inhibitors^62^. The best inhibitors were sulphamic acid, stannate, phenylarsonic acid, phenylboronic acid, and sulfamide (K_I_ values of 6.2–94 µM), whereas diethyldithiocarbamate, tellurate, selenate, bicarbonate, and cyanate were submillimolar inhibitors (K_I_ values of 0.71–0.94 mM). The halides (except iodide), thiocyanate, nitrite, nitrate, carbonate, bisulphite, sulphate, hydrogensulfide, peroxydisulfate, selenocyanate, fluorosulfonate, and trithiocarbonate showed K_I_ values in the range of 3.1–9.3 mM^62^. These prompted us to propose that BteCAι is probably a Zn^2+^- and not Mn^2+^-containing enzyme,^60^ as reported for diatom ι-CAs.^59^

### 2.2. Primary structure features of ι-CAs

LCIP63 and the homologs identified as bacterial ι-CAs (like BteCAι) show a primary sequence that completely differs from any previously identified CA-class.^59^^,^^60^ For example, LCIP63, at its N-terminal part, displays the presence of an endoplasmic reticulum signal peptide (of 22 amino acid residues) and a chloroplast signal peptide (of 34 amino acid residues)^59^. It is a multidomain protein with four, three, or two repeated domains, each of them homologous to the calcium/calmodulin-dependent protein kinase II Association Domain (CaMKII-AD)^59^. The CaMKII-AD belongs to the NTF2-like protein superfamily, which is a group of proteins, sharing a common fold identified for the first time in the structure of the rat NTF2 (Nuclear Transport Factor 2)^63^. Generally, the polypeptide chain of the bacterial ι-CAs present a pre-sequence of 19 or more amino acid residues at the N-terminal part and contains one or two repeated domains. The amino acid sequence is homologous to a group of proteins annotated as SgcJ/EcaC oxidoreductase family, with an unknown function. These proteins share a common structure with the NTF2-like superfamily, having a hydrophobic pocket that could constitute a putative substrate binding or catalytic active site.

**Figure 1.**
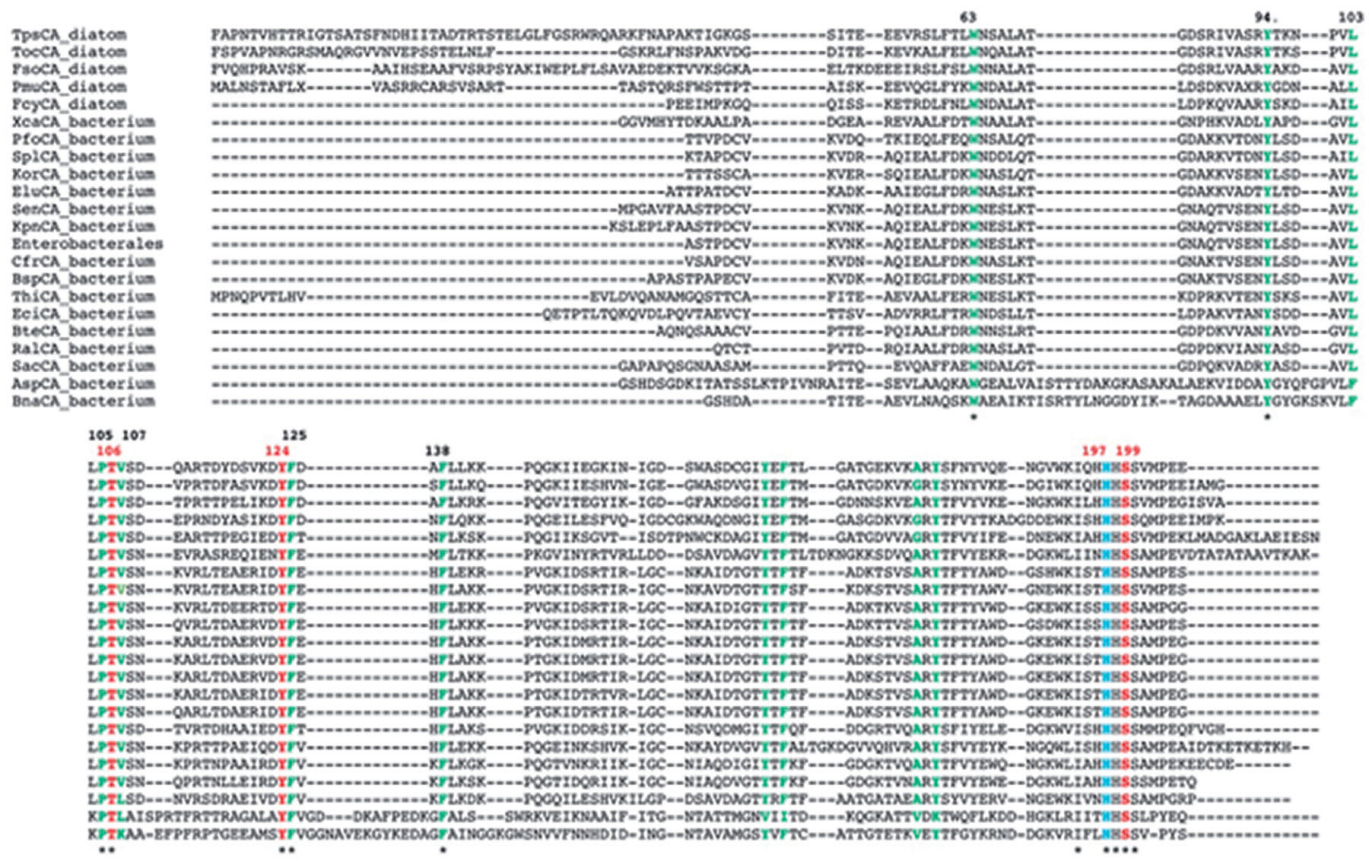
Multialignment of the ι-CA amino acid sequences from different species (bacteria, cyanobacteria, diatoms, and algae). In red, the putative residues of the catalytic triad (T106, Y124, S199); light blue colour, the H197 as the putative proton shuttle residue. In green are the other residues of the catalytic pocket (see Hirakawa et al. [64]). The putative motif (H)HHSS is the typical consensus sequence characterising the ι-CAs. The asterisk (*) indicates identity at all aligned positions. The multialignment was performed with MUSCLE, version 3.1. See Table 1 for the identification of the amino acid sequences used in the multialignment. The residue number system used refers to the AspCA enzyme.

Figure 1 reports the multialignment of ι-CA amino acid sequences from different species. It is evident that the ι-CAs do not show along the amino acid sequence the conserved residues essential for the catalytic mechanisms of all known CAs, such as the three histidine ligands (in α-, γ-, and δ-CAs), two histidine and one glutamine (of η-CA), or one histidine and two cysteines (from β-, ζ-, and θ-CAs). However, it is remarkable the presence in the C-terminal domain of all the amino acid sequences analysed and classified as ι-CAs (LCIP63-ιCAs and SgcJ/EcaC-ιCAs) of a consensus motif with the following residues: (H)HHSS, which seems to be a specific feature of ι-CAs (Figure 1).

## 3. ι-Cas (COG4337) with no metal ions within the catalytic site

Recently, a Japanese group identified novel CAs (acronym COG4337) encoded by the genome of the eukaryotic microalga *Bigelowiella natans* and the cyanobacterium *Anabaena sp.* PCC7120^64^. COG4337 homologs from eukaryotic organisms resulted in multidomain proteins formed of up to five domains, while the prokaryotic homolog genes encode only a single domain of about 160 amino acid residues^64^. The *Bigelowiella natans* and *Anabaena sp.* PCC7120 CAs (indicated here as BnaCA and AspCA) showed the typical consensus **HHSS** characterising the ι-CAs (Figure 1) mentioned above. They showed CO_2_ hydration activity, which was investigated by determining the WAU (Wilburn-Anderson Units). The enzyme activity of BnaCA and AspCA resulted in 7 and 37 times respectively lower than that obtained for a mammalian CA. Still, it was in the same range of the θ-CA from *Phaeodactylum tricornutum* and ι-CA from *T. pseudonana*^64^. Moreover, and this was a great surprise, both enzymes BnaCA and AspCA resulted to be catalytically active without the metal ion cofactor, unlike other reported ι-CAs as well as any other known CAs investigated so far^64^.

### 3.1. Phylogenetic analysis

**Figure 2.**
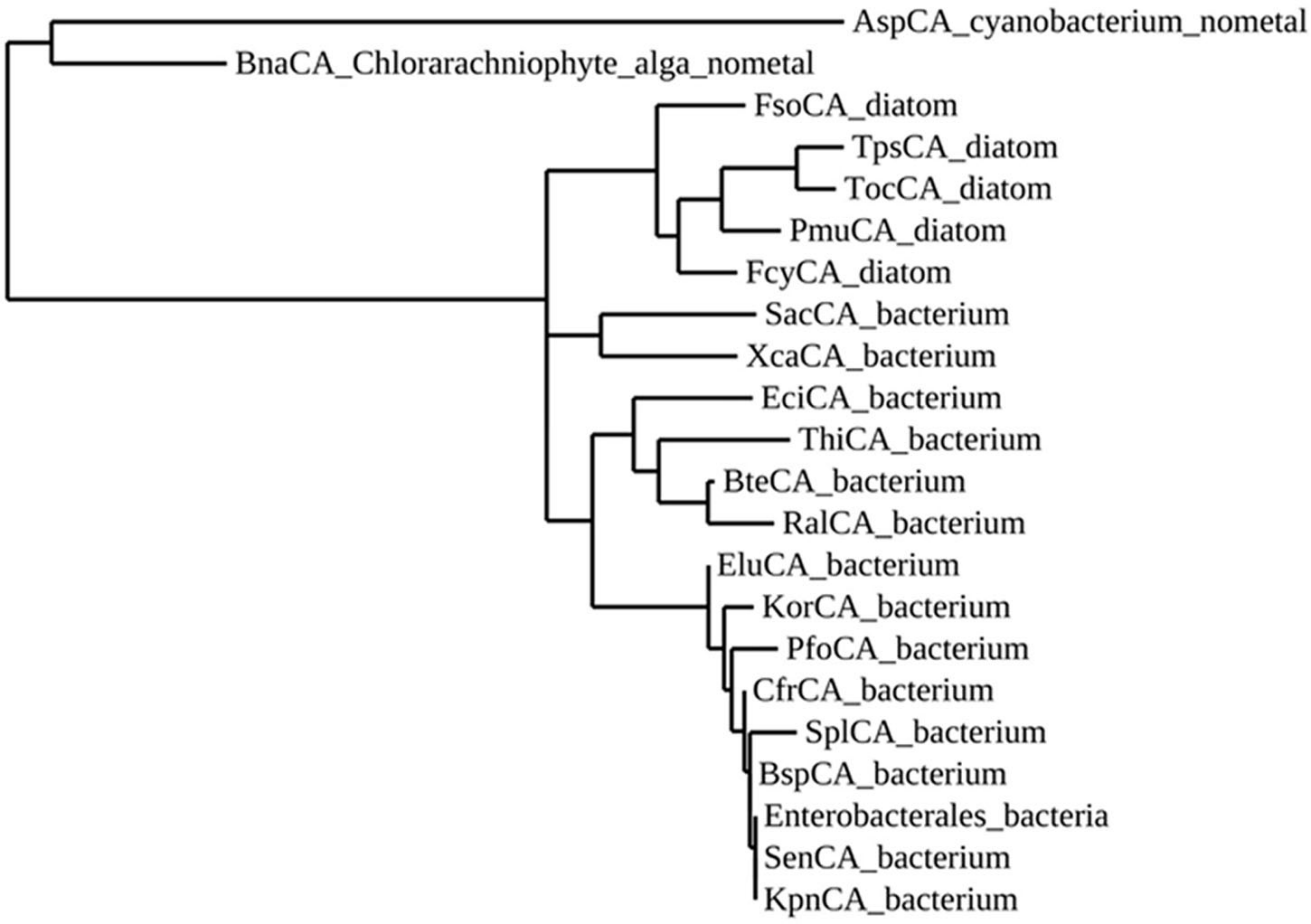
Phylogenetic analysis of ι-CAs from various organisms. The dendrogram was constructed using the ι-CA amino acid sequences reported in Table 1.

From the amino acid alignment of the two metal-free ι-CAs with those of ι-CAs from different other species, it is readily apparent that the main residues involved in the catalytic pocket of the two enzymes identified by Hirakawa et al. are completely conserved in all the polypeptide chains considered in this paper (Figure 1). A distinctive feature of the metal-free ι-CAs is the presence of an insertion absent in all the other presumably metal-containing ι-CAs (Figure 1). The analysis of the hallmarks present in the amino acid sequences is far from being exhaustive, as it does not consider all the amino acid substitutions that differentiate the novel metal-free-CAs from those of other ι-CAs. Hence, we have constructed a most parsimonious tree to better investigate the relationships of the novel ι-CAs identified by Hirakawa et al. with other ι-CAs from other species, such as diatoms and bacteria (Figure 2). In Table 1 is presented the information needed for the identification of the amino acid sequences used in the phylogenetic analysis. The two metal-free CAs appear closely associated with each other, as shown in the dendrogram in Figure 2. BnaCA and AspCA clustered in a branch distinct from all the other (presumably) metal-containing ι-CAs identified in diatoms and the bacterium species mentioned above. Thus, BnaCA and AspCA have several features typical of other ι-CAs, but in other aspects, they do not appear closely related to any other metal-ι-CAs. For this reason, they were annotated as a new subclass of the ι-CAs^64^. Moreover, Del Prete et al. demonstrated that ι-CAs clustered in a group closely associated with the bacterial γ-CAs^60^. Probably, from an ancestral γ-CA, during the evolution, a ι-CA originated developing a structural catalytic pocket, which evolved the CO_2_ hydration function, making possible the CO_2_ hydratase reaction without the metal ion cofactor as proposed by Hirakawa et al.^64^.

**Table 1.** Organisms, acronyms, and accession numbers of the amino acid sequences used in the phylogenetic analysis of ι-CAs.

Organism	Acronym	Accession number
*Anabaena sp.* PCC7120	AspCA_iota_nometal	7C5V_A
*Bigelowiella natans*	BnaCA_iota_nometal	7C5X_A
*Fistulifera solaris*	FsoCA_diatom	GAX12146.1
*Thalassiosira pseudonana*	TpsCA_Diatom	XP_002293761.1
*Thalassiosira oceanica*	TocCA_diatom	EJK63051.1
*Pseudo-nitzschia multistriata*	PmuCA_diatom	VEU38613.1
*Fragilariopsis cylindrus CCMP1102*	FcyCA_diatom	OEU14464.1
*Saccharothrix sp.*	SacCA_bacterium	WP_053720260.1
*Xanthomonas campestris*	XcaCA_bacterium	WP_011036063.1
*Ephemeroptericola cinctiostellae*	EciCA_bacterium	WP_114562659.1
*Thiotrichales bacterium*	ThiCA_bacterium	OYX05505.1
*Burkholderia territorii*	BteCA_bacterium	WP_063553346.1
*Ralstonia solanacearum*	RalCA_bacterium	WP_089190700.1
*Enterobacter ludwigii*	EluCA_bacterium	WP_074176950.1
*Kosakonia oryziphila*	KorCA_bacterium	WP_090137393.1
*Pragia fontium*	PfoCA_bacterium	WP_047780468.
*Salmonella enterica*	CfrCA_bacterium	ECP0341226.1
*Serratia plymuthica*	SplCA_bacterium	WP_064115041.1
*Buttiauxella sp.*	BspCA_bacterium	WP_183271195.1
*Enterobacterales*	Enterobacterales_bacteria	WP_003833285.1
*Salmonella enterica*	SenCA_bacterium	ECP0341226.1
*Klebsiella pneumoniae*	KpnCA_bacterium	WP_064115041.1

### 3.2. Three-dimensional structure analysis

**Figure 3.**
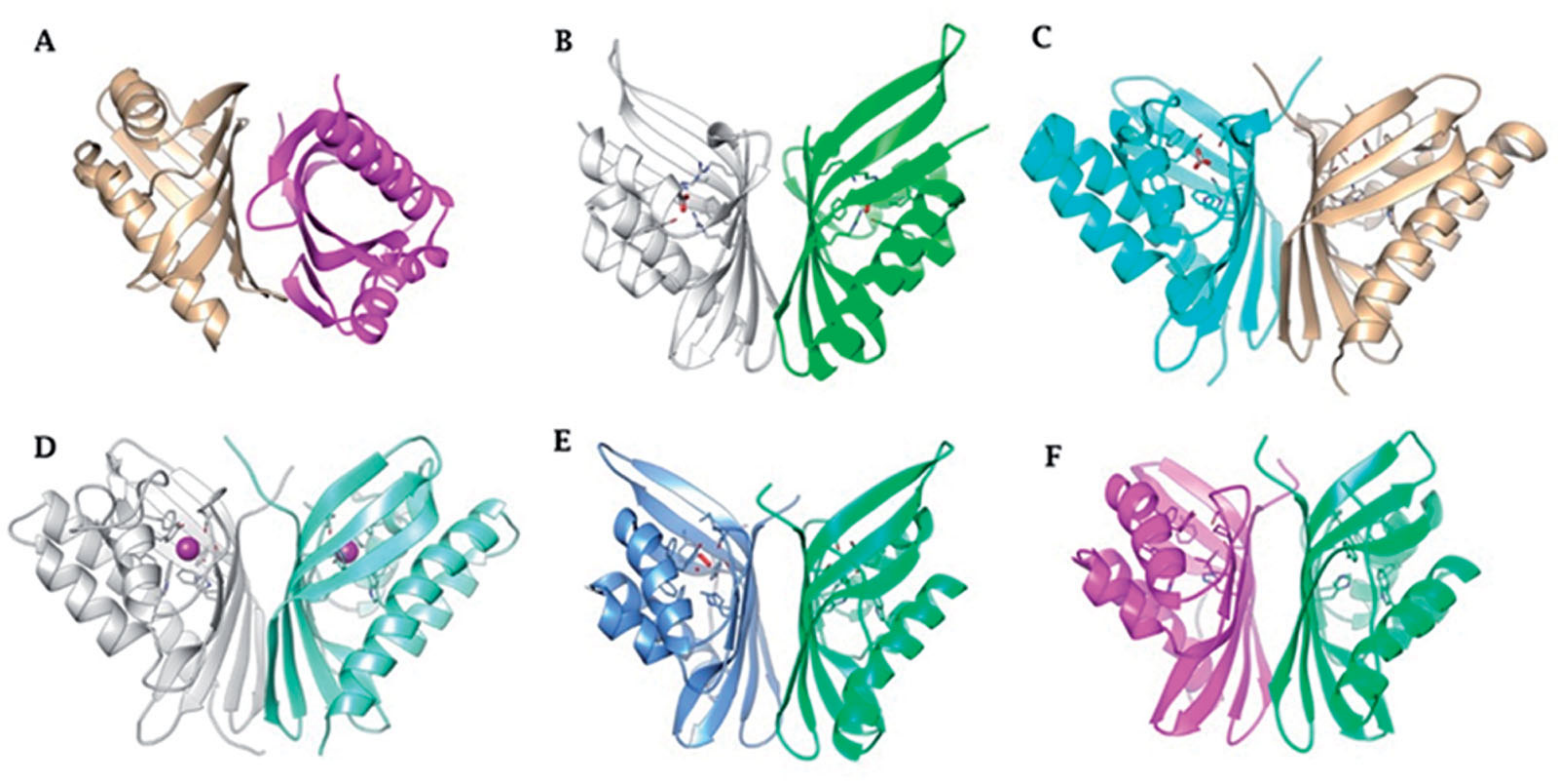
Ribbon view of (A) hNFT2 (PDB 1GY5), (B) CAMKII in complex with acetate (PDB 2W2C), (C) AspCA (ι-CA from *Anabaena* cyanobacterium) in complex with HCO_3_^-^ (PDB 7C5V), (D) BnaCA (ι-CA from microalga *Bigelowiella natans*) in complex with I^-^ (PDB 7C5Y), (E) XcaCA (ι-CA from *X. campestris*) in complex with putative HCO_3_^-^ (PDB 3H51), (F) homology model of BteCA (ι-CA from *B. territorii*) using XcaCA as template (51% identity).

X-ray crystallographic structures were obtained for COG4337 proteins in the presence of bicarbonate and the anion inhibitor iodide^64^. Figure 3 shows the three-dimensional structure of hNFT2 and CAMKII, two proteins belonging to the NTF2-like superfamily (Figure 3(A,B), respectively); the three-dimensional structure of AspCA and BnaCA (the novel ι-CAs identified by Hirakawa et al.^64^) obtained in the presence of bicarbonate and iodide anion (Figure 3(C,D), respectively), the crystal structures of an SgcJ/EcaC oxidoreductase identified in the genome of *Xanthomonas campestris* (Figure 3(E)) and BteCAι homology modelling (Figure 3(F)) generated with a fully automated protein homology modelling server SWISS-MODEL (https://swissmodel.expasy.org) and using template structure the homologous enzyme from *X. campestris*. Here, we want to stress that with the aid of protonography^65^, a biochemical technique used to identify the activity and the oligomeric state of CAs on SDS-PAGE, it has been demonstrated that BteCAι can be present as a dimer as shown by the obtained homology model^60^.

**Figure 4.**
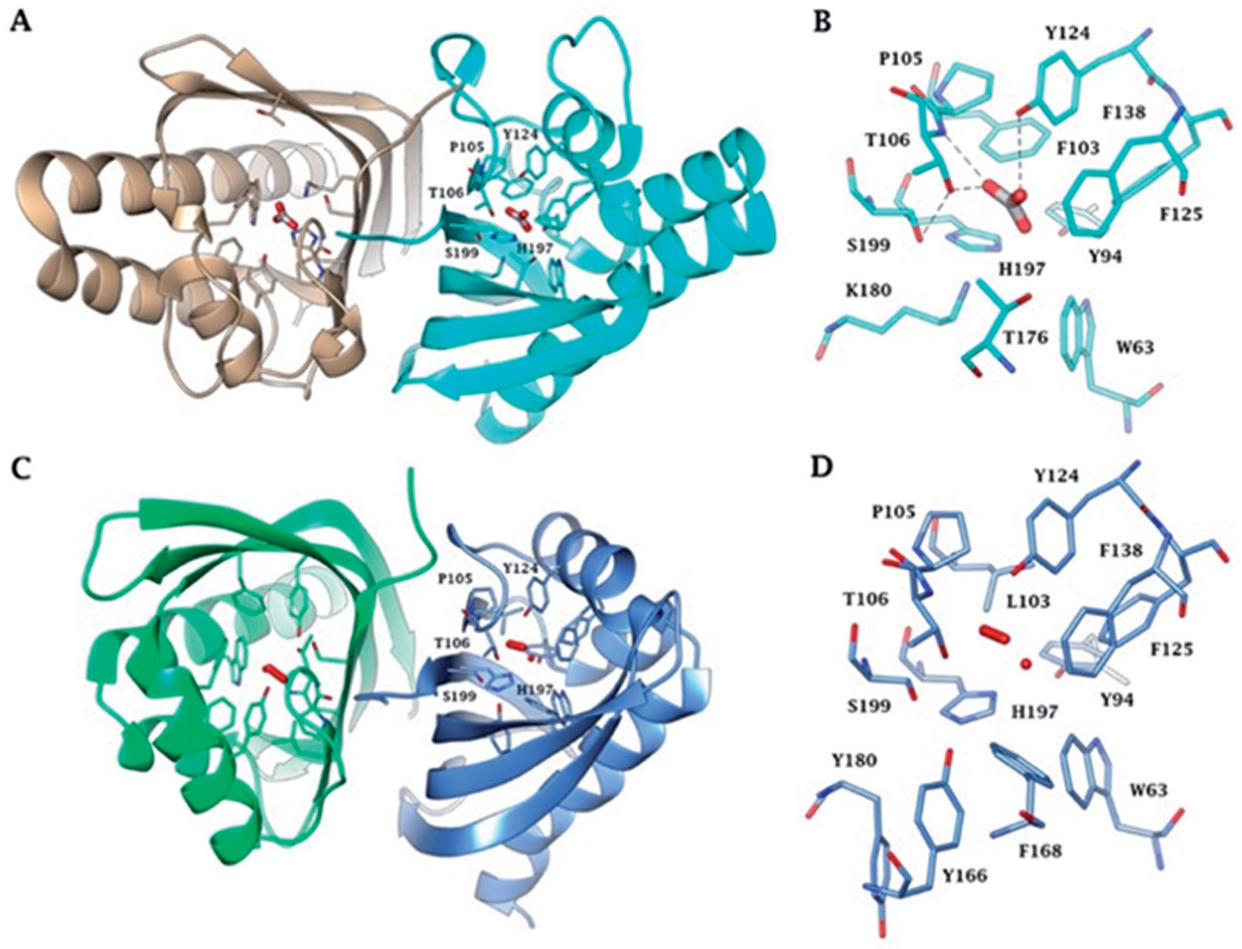
(A) Ribbon upper view and (B) binding site view of AspCA (ι-CA from *Anabaena* cyanobacterium) in complex with HCO3- (PDB 7C5V), (C) Ribbon upper view and (D) binding site view of XcaCA (ι-CA from *X. campestris*) in complex with putative HCO_3_^-^ (PDB 3H51). Residue numbers according to AspCA (PDB 7C5V).

Interestingly, all the enzymes reported in Figure 3 are homodimers with a very high degree of structural homology and belong to the NTF2-like family (Figure 3). The NTF2-like superfamily includes proteins widely found in both prokaryotic and eukaryotic organisms, which possesses highly versatile roles^63^. These proteins can perform a broad range of different functions because their three-dimensional folding form a channel that allows the introduction of differently molecular species^63^. The NTF2-like family represents a classic example of divergent evolution in which proteins have similar general structures but diverge significantly in their functions^63^, which often can be determined only from the biochemical analysis of the proteins. Our groups in fact recently demonstrated, with the aid of a stopped-flow spectrophotometer, that the bacterial amino acid sequence annotated as SgcJ/EcaC oxidoreductase and characterised by the motif (H)HHSS is in fact a CA, which acts as a good catalyst for the CO_2_ hydration reaction^60^. Intriguing, the homology model of BteCAι has a shape very similar to the crystal structure of the AspCA and BnaCA obtained with a bicarbonate molecule or iodide ion located inside the cone-shaped barrel, respectively. The most crucial evidence of AspCA and BnaCA structure is that the electron densities corresponding to metals were not detected in the structure cavity of both enzymes, confirming that the catalytic activity of these two enzymes is not dependent on the presence of the metal ion Figure 4. Indeed, the residues of the catalytic pocket which are involved in the binding of bicarbonate and ion iodide are evidenced in Figure 4. We want to stress that the model of BteCAι presented in 2020 did not allow us the insertion of the zinc ion in the interface of the two monomers to make possible the metal coordination with two histidines of a monomer and one histidine of the other monomer^60^. However, it is also true that the BteCAι enzyme catalytic activity was observed by adding Zn^2+^ as described by Del Prete et al.^60^. On the other hand, it may be possible that the zinc has not a catalytic but a structural function in the bacterial ι-CAs.

**Figure 5.**
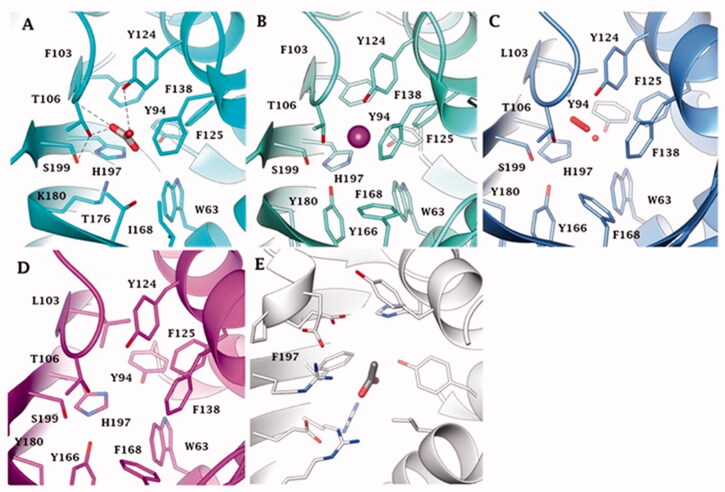
Binding site view of (A) AspCA (ι-CA from *Anabaena* cyanobacterium) in complex with HCO_3_^-^ (PDB 7C5V), (B) BnaCA (ι-CA from microalga *Bigelowiella natans*) in complex with I^-^ (PDB 7C5Y), (C) XcaCA (ι-CA from *X. campestris*) in complex with putative HCO_3_^-^ (PDB 3H51), (D) homology model of BteCA (ι-CA from *B. territorii*) using XcaCA as template (51% identity), (E) CAMKII in complex with acetate (PDB 2W2C). Residue numbers according to AspCA (PDB 7C5V).

Figure 5 reports the metal-free ι-CAs, the ι-CAs from *B. territorii*, and the CaMKII (NTF2-like superfamily) binding pocket. The three-dimensional arrangement of AspCA and BnaCA evidenced a catalytic site with a shape of a cone whose cavity is formed by hydrophilic (Thr, Ser, His, Lys, and Tyr) and hydrophobic (Trp and Phe) residues (Figure 4). Through the experiments of point mutation analysis, essential amino acids of the COG4337 catalytic pocket have been highlighted, and a putative catalytic mechanism for the CO_2_ hydration reaction has been proposed for these metal-free CAs (Figure 4). In the known metallo-CAs, such as the human isoforms hCA I and hCA II, the initial step of the reaction involves the deprotonation of H_2_O in the active site to generate the nucleophile OH^-^ ion. It attacks the CO_2_, producing the HCO_3_^-^; and the gatekeeper residues (Thr199 and Glu106) accept a hydrogen bond from the zinc-bound water, while the proton shuttle residue (His64) has the function to push away the protons from the active site. In the BnaCA and AspCA, metal-free CAs, the hydroxyl groups of the Thr106, Tyr124, and Ser199, present in the catalytic site, were proposed to be involved in the deprotonation of the active site water. The function of the proton shuttle is probably mediated by the H197 or the Tyr positioned on the molecular surface of the enzyme (Figure 4). Hirakawa et al. assumed that the CO_2_ binding site could be the hydrophobic part of the catalytic pocket. Interestingly, the metal-free ι-CA probably does not exhibit the reversible dehydration reaction^64^, of bicarbonate to CO2, which might be a peculiar feature of the metal free ι-CA, as all other known metallo-CAs catalyse both the CO_2_ hydration as well as bicarbonate dehydration reactions^2^^,^^7^^,^^8^.

## 4. Conclusions

The recent report of a metal-free CA belonging to the ι-class^64^ and a very interesting proposal for the catalytic mechanism of these enzyme for the CO_2_ hydration reactions, prompted us to investigate in detail the phylogenetic relationship, primary, secondary and tertiary structures of the other two ι-CAs investigated in detail: the presumably manganese-containing enzyme from a diatom (*T. pseudonana*)^59^, and the bacterial, presumably zinc-enzyme from *Burkholderia territorii*^60^. This analysis however also included many such sequences from other organisms, which have not yet been characterised in detail. It was thus observed that ι-CAs possess a rather relevant structural homology with the NTF2-like family of proteins, which have a great variety of functions and physiological roles^63^. Furthermore, the residues that have been proposed to be involved in the CO_2_ hydration reaction of the metal-free ι-CAs, were observed to be conserved in all sequences of such enzymes present in diatoms and bacteria. Coupled to the fact that by computational techniques we were unable to position zinc ions in the model of BteCAι, although we have determined the presence of one mole of Zn^2+^ per polypeptide chain of this protein (using atomic absorption spectrophotometry)^60^ prompts us to hypothesise that probably in all ι-CAs the metal ions may not have a catalytic but a structural function (although in the X-ray crystal structure of AspCA no metal ions were present). It is also possible that the reported zinc or manganese ions necessary for the catalytic activity of some of the ι-CAs is an artefact due to the ubiquity of some metal ions present in traces in most reagents, solvent, glass, etc. However, we wish to stress here, the results reported by Jenssen et al.^59^ and Del Prete et al.^60^ are valid, even if those enzymes are metal free CAs. In fact, Hirakawa et al.^64^ reported also adducts with anion inhibitors of AspCA (iodide and bicarbonate), and such an inhibition effect was also reported with various anions (inorganic and organic ones) for the presumably metal-containing ι-CAs^59–62^. Thus, future work is needed to establish whether all ι-CAs are metal free, or whether some of them may use manganese or zinc ions within their active site, and the subclass reported by Hirakawa et al.^64^ is a just a minority of such enzymes.
